# The effectiveness of health coaching, home blood pressure monitoring, and home-titration in controlling hypertension among low-income patients: protocol for a randomized controlled trial

**DOI:** 10.1186/1471-2458-9-456

**Published:** 2009-12-10

**Authors:** Heather Bennett, Kelsey Laird, David Margolius, Victoria Ngo, David H Thom, Thomas Bodenheimer

**Affiliations:** 1 Department of Family and Community Medicine, University of California, San Francisco (UCSF), 1001 Potrero Ave, Building 80/83, San Francisco, CA 94110, USA

## Abstract

**Background:**

Despite the many antihypertensive medications available, two-thirds of patients with hypertension do not achieve blood pressure control. This is thought to be due to a combination of poor patient education, poor medication adherence, and "clinical inertia." The present trial evaluates an intervention consisting of health coaching, home blood pressure monitoring, and home medication titration as a method to address these three causes of poor hypertension control.

**Methods/Design:**

The randomized controlled trial will include 300 patients with poorly controlled hypertension. Participants will be recruited from a primary care clinic in a teaching hospital that primarily serves low-income populations.

An intervention group of 150 participants will receive health coaching, home blood pressure monitoring, and home-titration of antihypertensive medications during 6 months. The control group (n = 150) will receive health coaching plus home blood pressure monitoring for the same duration. A passive control group will receive usual care. Blood pressure measurements will take place at baseline, and after 6 and 12 months. The primary outcome will be change in systolic blood pressure after 6 and 12 months. Secondary outcomes measured will be change in diastolic blood pressure, adverse events, and patient and provider satisfaction.

**Discussion:**

The present study is designed to assess whether the 3-pronged approach of health coaching, home blood pressure monitoring, and home medication titration can successfully improve blood pressure, and if so, whether this effect persists beyond the period of the intervention.

**Trial Registration:**

ClinicalTrials.gov identifier: NCT01013857

## Background

Hypertension remains a leading cause of preventable death despite the availability of evidence-based treatments. Even small reductions in high blood pressure have major impacts in clinical outcomes and health care spending: a 2 mm Hg decrease in systolic blood pressure (SBP) or diastolic blood pressure (DBP) significantly reduces risk of stroke, coronary heart disease, and mortality from vascular causes [1]. In the United States, over thirty percent of adults have hypertension, yet of these, only one third achieve normal blood pressure [2,3]. The discrepancy between controlled and uncontrolled hypertensive patients is even wider among ethnic minorities and low-income populations [4].

Poor blood pressure control is frequently attributed to low rates of medication adherence. Indeed, several studies have shown that only fifty to seventy percent of patients take their blood pressure medications correctly [5]. One explanation for the high rate of non-adherence is that many patients lack an understanding of the disease and its medications. The asymptomatic nature of hypertension contributes to this lack of understanding [6]. Many patients believe that hypertension is intermittent and can be best treated with non-pharmacological therapies such as stress relief or home remedies [3].

In the traditional primary care setting, patients comprehend as little as half of what their physicians convey during the appointment [7]. Yet improved health education by itself does not improve blood pressure control or medication adherence [5]. Encouraging patients to become active participants in their care, and imparting them with the skills and confidence needed for such active participation has been shown to improve blood pressure control [8,9]. Health coaching is an innovation designed to provide patients with self-management support, arming them with the information, skills and confidence needed to become active participants in their care.

Lack of treatment intensification by clinicians may be equally, if not more, important than medication adherence in attributing to poor control in hypertensive patients. Higher rates of treatment intensification are associated with improved blood pressure control [10]. However, one study found that antihypertensive medications were increased in only thirteen percent of visits in which blood pressure was elevated [11]. In a separate study, lack of timely medication intensification (the physician factor) contributed more to poor blood pressure control than low medication adherence (the patient factor) [12]. The failure to intensify treatment when appropriate has been labeled "clinical inertia" [13].

In addition to providing patients with self-management support, health coaches, using physician-created algorithms, can relay physician advice on medication intensification to patients without a physician visit. In this way, health coaches can address both the patient and physician factors associated with poor blood pressure control.

A series of publications have shown that ambulatory blood pressures better predict cardiovascular risk compared to clinic blood pressure readings [14]. One study attempted to overcome clinical inertia by giving patients the ability to titrate their own medications based on their home blood pressure measurements [15]. At 8 weeks, patients given the option to self-monitor blood pressures and titrate medications according to an algorithm had significantly lower blood pressures compared to patients in the office-based (usual) care group who neither measured home blood pressures nor titrated their own medications. A second study evaluated home blood pressure monitoring and monthly pharmacist phone calls to reduce high blood pressure [16]. The pharmacists reported abnormal values to the patient's primary care physician (PCP) along with recommendations for a titration. The blood pressure in the intervention group was improved over the usual care group and this improvement was associated with increased modifications of treatment regimens. A third study concluded that self-monitoring was only associated with better blood pressure control when it contributed to the proactive communication of abnormal values to the clinician [8]. These studies indicate that improved blood pressure can be achieved when home blood pressure monitoring allows patients to be part of clinical decision-making.

Based on the findings cited in the previous paragraphs, the present study was designed to combine health coaching, home blood pressure monitoring, and home-titration of antihypertensive medications to overcome the tri-fold barriers of inadequate health education, poor medication adherence and clinical inertia. This study is the first to use health coaches with some or no formal clinical training in place of RNs, pharmacists, or physicians for this purpose.

## Materials/Design

### Objectives

Our study investigates whether weekly telephone coaching on home blood pressure monitoring and medication adherence results in improved blood pressure control during a 6 month coaching period. Furthermore, we aim to determine whether home-titration of antihypertensive medications further increases this benefit, and whether these effects are sustained 6 months after the coaching period has ended.

### Study Design

Hypertensive patients are randomly assigned to one of two groups:

1) Control group: Patients receive weekly health coaching phone calls on home blood pressure monitoring, medication understanding, and medication adherence.

2) Active group: Patients receive weekly health coaching phone calls on home blood pressure monitoring and medication understanding and adherence, as well as assistance with home-titration of their antihypertensive medications.

A passive control group, receiving usual care, will be evaluated by retrospective chart review upon the completion of the study.

### Ethics

Ethical approval to conduct this study has been granted by the Committee on Human Research at the University of California, San Francisco (approval number H40013-33128-01).

### Study population and site

All participants are low-income English, Spanish, Cantonese, or Vietnamese speaking patients who receive primary care at the Family Health Center in San Francisco General Hospital. The Family Health Center is a primary care clinic in a teaching hospital.

### Inclusion criteria

Patients are eligible for the study if they have a documented blood pressure greater than or equal to 145/90 on two or more separate visits in the past year. SBP must be greater or equal to 145 or DBP greater or equal to 90 on both readings. The two readings can include an elevated reading taken on the day that the patient is enrolled. Eligible patients are not required to carry a diagnosis of hypertension or be prescribed antihypertensive medications at enrollment. Patients must speak Cantonese, English, Spanish, or Vietnamese.

### Exclusion criteria

Patients are excluded from the study if they are less than 30 years of age, their most recent creatinine is greater than 1.5 mg/dl, they have class III or IV heart failure, their life expectancy is less than one year or they have reduced cognitive capacity as determined by the patient's PCP. Patients with reduced cognition are included in the study if they have a known caregiver, such as a family member, capable of assisting the patient with home blood pressure monitoring and medication titration, and speaking with the health coach each week.

### Identification and recruitment of participants

San Francisco's Department of Public Health electronic patient registry was searched for patients that: (1) are assigned to a PCP at San Francisco General Hospital's Family Health Center, (2) have a diagnosis of hypertension, (3) are greater than or equal to thirty years of age, (4) have no documented serum creatinine reading greater than 1.5 mg/dl, and (5) speak Cantonese, English, Spanish, or Vietnamese.

PCPs are given the list of their screened patients and are asked to exclude patients whom they believed would be unable to monitor their blood pressure or follow medication titration instructions due to physical or cognitive disability, psychiatric illness, or other reasons.

Additionally, patients with elevated blood pressures in the clinic are screened and recruited. Clinic staff or PCPs alert the study's health coaches if any patient has a blood pressure greater than 145/90 measured during intake. Health coaches review the chart to see if the patient met the eligibility criteria, including a blood pressure greater than 145/90 on a separate visit in the previous year. If not referred directly by the PCP, the health coach approaches the patient's PCP to see if the patient was eligible in terms of cognitive ability and life expectancy.

### Patient enrollment, training, and randomization

Eligible patients are approached by a language-concordant health coach at the time of their primary care appointment and interviewed to ensure that they: (1) plan to continue coming to the Family Health Center for the next year, (2) have a telephone, (3) are willing to check their blood pressure at least twice a week, and (4) are willing to learn how to change their blood pressure medicines from home if enrolled in the active group. If patients answer yes to all of the above, they are given IRB-approved informed consent for enrollment in the study.

Patients are given a home blood pressure monitor (Omron model HEM-711AC) and trained in its use. They are also given a calendar logbook in which to record their blood pressures and heart rates. In order to ensure comprehension, health coaches request that patients "teach back" the process of checking their blood pressure and recording the values in their logbook.

At the completion of the training and consent process, health coaches and patients learn the patient's randomly generated enrollment assignment to either the active or control group. The health coach also confirms with the patient any currently prescribed antihypertensive medications and dosages.

### Sample size calculation

Sample size calculation for detecting change in systolic blood pressure as the primary outcome used a standard deviation of 15 mm Hg was used based on prior studies that have reported standard deviations ranging from 10 to 20 mm Hg. 15 mm Hg was also the standard deviation used to calculate sample size for a recent study [17]. To be able to detect an absolute difference of at least 5 mm Hg in SBP at a 2-tailed significance level of .05, and a power of 82%, 150 participants in both groups are needed. Even allowing for a substantial drop out of 30%, there is still an 83% power to detect a true effect size of 6 mm Hg.

### Data analysis

Chi-square tests (for dichotomous and categorical variables), t-test (for continuous, normally distributed variables), and non-parametric tests (e.g., Mann-Whitney-U) will be used to compare the two groups with respect to demographic characteristics, blood pressure at the time of enrollment, number and type of medications, length of time since diagnosis of hypertension, number of clinic visits in the past 12 months, primary language, and co-morbidities. The primary outcome, change in systolic blood pressure at 6 months, will be compared between the two groups using a simple t-test against the usual null hypothesis of no difference. Analyses will be performed to assess the impact, if any, of adjusting for any differences in group characteristics using multivariate analysis of variance (MANOVA). If the unadjusted (t-test) and adjusted (analysis of variance) results differ, the analysis of variance approach will take precedence.

Secondary outcomes will include difference in diastolic blood pressure, the proportion of patients achieving BP < 140/90, and proportion of patients reporting medication side effects (mild, moderate, and severe). These will be examined as per primary outcomes. Further analyses will be conducted to look for evidence of effect modification by pre-specified subgroups: starting systolic BP (≥ 160 vs. <160), by English as primary language (Y/N), and age (< median versus ≥ median). These analyses will be reported as exploratory.

### Health coaches

Health coaches are employees and volunteers at the UCSF Department of Family and Community Medicine. Of the 10 health coaches, eight have no formal clinical training. Health coaches attend at least two training sessions co-taught by a clinician and medical student who are part of the study team. Training topics include health coaching, basic hypertension pathophysiology, antihypertensive medication therapy, side effects (including red-flag symptoms), home blood pressure monitoring, standard home titration algorithms, emergency protocols, and patient confidentiality. Each health coach tracks a panel of five to thirty patients concurrently. The two principal investigators - both physicians - are available to answer questions about clinical cases and study protocols, as was occasionally necessary throughout the study.

### Telephone coaching

Health coaches telephone each of the patients in their panel on a weekly basis, and completed an encounter form for each phone call. If available, health coaches also meet patients face-to-face before or after a PCP appointment at the Family Health Center during the 6 month coaching period. Health coaches also arrange for patients to meet specifically with them in clinic if they were having trouble using their blood pressure monitor or if face-to-face medication education was needed.

Data recorded during weekly telephone health coaching encounters includes: (1) usual, highest and lowest recorded BP for that week; (2) current BP (only collected if BP was not monitored regularly that week or if readings were highly variable); (3) usual, highest and lowest heart rate (HR); (4) current heart rate (only collected if heart rate not monitored regularly or if readings were highly variable).

In addition, patients are asked how many days they measured their blood pressure that week, how many days they miss taking an antihypertensive medication, whether they are having any side effects from these medications, and whether they have had any major events such as falls or unplanned visits to the hospital emergency room. Patients in both groups are encouraged to make an action plan with their health coach for improving their blood pressure by the next week. Action plans involve health coaches and patients agreeing on a concrete course of action to move toward a general goal of improved blood pressure [18]. Patients in the control group are scheduled for appointments if they feel they need medication intensification. For patients in the active group, the health coach and patient have the option to home-titrate blood pressure medications.

### Home-titration of antihypertensive medication

**Figure 1:**
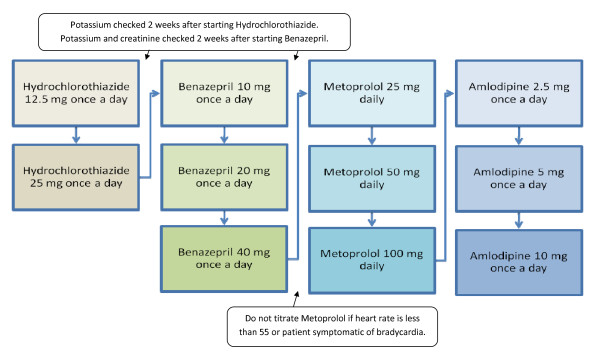
**Home-titration algorithm for antihypertensive medications**.

Antihypertensive medications were titrated using an algorithm adapted from Kaiser Permanente (see figure 1). Patients and health coaches came to a shared decision to titrate if: (1) blood pressures are above goal, (2) patients have good medication adherence of currently prescribed medications, and (3) patients do not report any side effects that would warrant further investigation before increasing medication dosage. Health coaches communicate with one of the principal investigators of the study to request that the medication change be made in the electronic medical record and faxed to the patient's pharmacy. The algorithm ensures potassium and creatinine levels are checked 2 weeks after starting specific medications, as guidelines recommend [2]. Patients' PCPs are informed once the medication titrations are made.

### Outcome measures

The primary outcome is systolic blood pressure measured at 0 months (enrollment), 6 months (end of coaching intervention period) and 12 months. Secondary outcome measures include diastolic blood pressure, side effects, adverse events, and patient and provider satisfaction.

### Qualitative evaluation

After 6 months of participation in the study, patients are asked to respond to a number of questions regarding their experience working with their health coach, including satisfaction with the health coach, with monitoring blood pressure at home, and - if applicable - with changing blood pressure medicines without visiting their doctor.

## Discussion

Advancements in antihypertensive medications offer the possibility of dramatically reducing the incidence of heart attacks and strokes worldwide. Evidence-based guidelines for hypertension are widely distributed. However, persistently poor levels of blood pressure control can only be addressed by confronting the triple barriers of poor patient understanding, low medication adherence, and clinical inertia. The current study is based on the concept that these three barriers result in large part from PCPs lacking time to adequately manage common chronic illnesses [19]. The involvement of non-physician members of the primary care team - in this study, health coaches - is necessary to achieve better population-wide blood pressure control.

In our study, we trained health coaches with some or no prior medical training to discuss blood pressure goals and antihypertensive medications with patients over the phone, thus eliminating the time constraint of the fifteen-minute physician visit. In one group, health coaches titrate medications according to an algorithm, thus preventing long revisit intervals from delaying appropriate medication intensification.

We hypothesize that both arms of the intervention will increase the number of patients with controlled hypertension at six and twelve months relative to the passive control group. We further hypothesize that patients in the active group, who are coached on home-titration, will achieve blood pressure control that is equal or better than that of patients in the control group, with no home-titration of antihypertensive medications.

## Competing interests

The authors declare that they have no competing interests.

## Authors' contributions

HB, VN, DT, and TB conceived the study. DT planned the statistical analysis, and conducted sample size calculation. All authors were responsible for the drafting of this paper and approved the final manuscript.

## Pre-publication history

The pre-publication history for this paper can be accessed here:

http://www.biomedcentral.com/1471-2458/9/456/prepub
